# The Influence of Slide Burnishing on the Technological Quality of X2CrNiMo17-12-2 Steel

**DOI:** 10.3390/ma17143403

**Published:** 2024-07-10

**Authors:** Tomasz Dyl, Dariusz Rydz, Arkadiusz Szarek, Grzegorz Stradomski, Joanna Fik, Michał Opydo

**Affiliations:** 1 Department of Marine Maintenance, Faculty of Marine Engineering, Gdynia Maritime University, Morska Street 81-87, 81-225 Gdynia, Poland; t.dyl@wm.umg.edu.pl; 2 Faculty of Production Engineering and Materials Technology, Czestochowa University of Technology, 19 Armii Krajowej Av., 42-201 Czestochowa, Poland; dariusz.rydz@pcz.pl (D.R.); michal.opydo@pcz.pl (M.O.); 3 Faculty of Mechanical Engineering and Computer Science, Department of Technology and Automation, Czestochowa University of Technology, 21 Armii Krajowej Av., 42-201 Czestochowa, Poland; arkadiusz.szarek@pcz.pl; 4 Faculty of Science and Technology, Jan Dlugosz University in Czestochowa, Armii Krajowej Street 13/15, 42-200 Czestochowa, Poland; j.fik@ujd.edu.pl

**Keywords:** slide burnishing, degree of relative hardening, roughness, microstructure

## Abstract

Metal products for the metallurgical and machinery industries must meet high requirements in terms of their performance, including reliability, accuracy, durability and fatigue strength. It is also important that materials commonly used to manufacture such products must meet specific requirements. Therefore, various techniques and technologies for modifying the surface layer are becoming more and more widely used. These include burnishing, which may be dynamic or static. This article studies the process of slide burnishing of surfaces of cylindrical objects. The burnishing was performed using a slide burnisher with a rigid diamond-tipped clamp on a general-purpose lathe. The tests were performed for corrosion-resistant steel X2CrNiMo17-12-2. The aim of the research was to determine the impact of changes in burnishing conditions and parameters—feed rate, burnisher depth and burnishing force at a constant burnishing speed—on the surface roughness and hardness. Additionally, the microstructure was assessed in the critical areas: the surface and the core. Another phenomenon observed was surface cracking, which would be destructive due to the occurrence of indentation. In the paper, it was stated that the microstructure, or rather the grains, in the area of the surface layer was oriented in the direction of deformation. It was also observed that in the area of the surface layer, no cracks or other flaws were revealed. Therefore, slide burnishing not only reduces the surface roughness but hardens the surface layer of the burnished material.

## 1. Introduction

Contemporary manufacturers strive to offer more and more reliable products. They are constantly improving their methods of producing materials and finishing end products. Because they are trying to meet increasing requirements while keeping production costs low, we can see rapid development in various fields of technical science, including material engineering, which improves both manufacturing processes and the functional properties of their outcomes. New corrosion-resistant steel grades are an example of such achievements. The origin of these materials dates back to the beginning of the 20th century. Metallurgists in Sheffield (Great Britain) discovered that steel with an approximately 13% admixture of chromium was more resistant to electrochemical corrosion. The New York Times mentioned the production of the first “non-rusting steel” in 1915 [1]. However, a patent for chrome–nickel stainless steel was filed and accepted by German Kaiserliches Patentamt earlier in 1912 [2]. Chrome–nickel steels of the “18-8” type were developed in the 1920s. Stainless steels are today a group of materials that are very important from an economic point of view. Due to the well-mastered technology of stainless steel production, an important aspect is reducing costs while staying in control of the functional properties of the material. The steel industry is committed to continuous improvement and modernization of its production technologies to meet the increasingly higher requirements of the market. However, quality improvement should not be followed by an excessive increase in product prices. The need to balance these aspects has been a strong driver for development in many fields of science including mechanical and material engineering. This ongoing process is aimed mainly at developing newer technologies to improve production processes and their outcomes [3,4,5,6,7,8]. One of them is burnishing, which has slightly sunk into oblivion.

Innovative products of the metallurgical and machinery industries must meet high requirements in terms of their performance, especially reliability, precision and durability. It is also important that the materials commonly used for the manufacture of machine components—such as uniform and stepped shafts, drive rotors, etc.—should have specific features [9,10]. Generally, burnishing can be dynamic or static [10,11,12,13]. The former method involves hitting the workpiece with a tool, which exerts variable pressure on the metal surface. In the case of static burnishing, the tool stays in contact with the surface, so the pressure is constant.

The main criteria used to distinguish between burnishing methods include the following [10,13,14,15]:

− The number and shape of the burnishing elements of the tool;
− The timing of contact between the tool and the workpiece: steady vs. pulsed;
− The mode of applying pressure to the workpiece: rigid or elastic;
− The type of friction between the tool and the workpiece.

**Figure 1:**
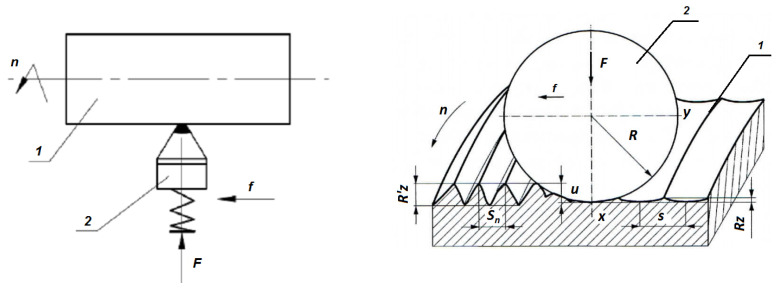
Slide burnishing arrangement [7]—(1) workpiece, (2) tool.

The slide burnishing process described in this article is a specific type of static burnishing. There is sliding friction between the burnishing tip of the tool and the workpiece. The distinguishing feature of slide burnishing is the slippage between the burnishing tip and the machined surface [7,8,11,16]. An example configuration of the slide burnishing process is shown in Figure 1. The burnishing force should be optimal for each type of burnishing treatment. An excessive force may cause surface flaking—a sharp increase in roughness. However, if the force is too small, it will not be able to smoothen the surface [7].

Figure 1 shows the slide burnishing for smoothness with a spherical-tipped tool, where F—burnishing force [N], f—feed rate [mm/rev], n—rotational speed [rpm], R′z—surface roughness ridge height before burnishing, Rz—surface roughness ridge height after burnishing, R—tool circular cross-section radius, u—burnisher depth, Sn—surface roughness ridge pitch before burnishing and s—surface roughness ridge pitch after burnishing.

**Figure 2:**
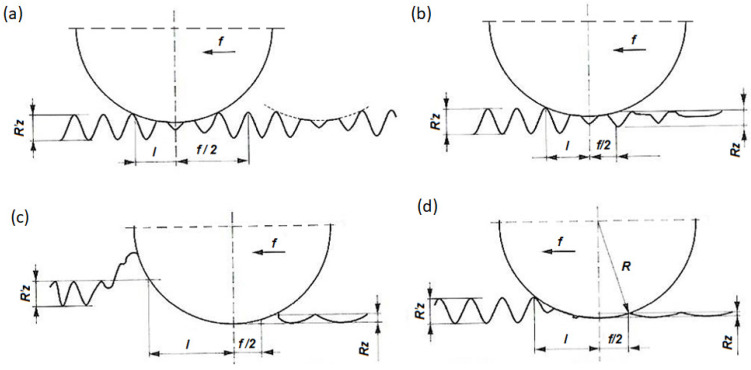
Slide burnishing performance as a function of feed rate and burnisher depth [10]: (**a**) feed rate larger than the contact length and small burnisher depth, (**b**) feed rate smaller than the contact length and small burnisher depth, (**c**) feed rate smaller than the contact length and large burnisher depth, (**d**) feed rate smaller than the contact length and burnisher depth. f—feed rate [mm/rev], l—contact length [mm], R′z—surface roughness ridge height before burnishing, Rz—surface roughness ridge height after burnishing, R—tool circular cross-section radius.

Figure 2 shows the influence of the feed rate and burnisher depth on the surface roughness after slide burnishing. The differences between the roughness profiles depend on the feed rate and burnisher depth. In the first case (a), the feed rate is larger than the length of contact of the tool face with the machined surface (in the tool travel direction). Areas that are not fully plastically deformed remain on the machined surface. However, the surface ridge height in these areas is the same as before the burnishing. Such machining conditions are unacceptable. In the second case (b), where the feed rate is lower than the contact length and the burnisher depth is small (less than half the roughness ridge height), the surface after burnishing is smoother than before (but not the best achievable). Therefore, the metal extruded by the tool from the ridges does not fully fill the valleys. In the third case (c), where the feed rate is lower than the contact length and the burnisher depth is large (sufficient to form a “headwave” and a “tailwave” of plasticized metal), the surface ridge height and shape are different and can be predicted using geometric and kinematic relationships. Also, this approach to machining is undesirable because, again, the result is less than optimal. Case (d) represents the conditions optimal for surface smoothing: the feed rate is smaller than the contact length and the burnisher depth is such that the ridges are flattened and their material is pressed into the valleys without any tailwave. This scenario may be considered optimal for slide burnishing. The ultimate surface quality strongly depends on the careful selection of the machining parameters. Based on the literature, it was determined that a feed rate (f) in the range of 0.03–0.08 mm/rev and a burnishing force (F) in the range of 20–200 N are recommended [7,9,10,11,12,14].

When designing a process for the production or reworking of machine elements, we should choose a burnishing method, machining conditions, geometries and number of burnishers depending on the method of applying pressure to the surface, which can be elastic or rigid. The reliability of machines and devices is a very important issue and is discussed in many studies. As an example, they can be used to repair individual components of ship machinery. Such work is often undertaken during long cruises. Burnishing processes are also used when restoring the outer surfaces of rolls intended for plastic forming. The selection of burnishing parameters is made based on preliminary calculations of burnishing unit forces and pressures, the results of experimental tests of materials with similar properties, general-purpose nomograms and specialized standards [15,16,17]. In the absence of sufficiently credible calculation formulas and nomograms, and for burnishing combined with cutting, the selection of burnishing conditions is made based on preparatory tests [16,17].

The burnishing process intended to minimize surface roughness should use quite a strong burnishing force. The burnishing rate and the feed rate should be relatively small. The burnishing process itself, which is intended to strengthen the surface layer of machine components, among others, by increasing hardness, should be characterized by the use of high burnishing forces at low feed rate and burnishing speed values. The burnishing force should be optimal for each burnishing process type. Applying a force stronger than optimal may result in surface flaking, accompanied by a sharp increase in roughness. On the other hand, a force weaker than optimal cannot provide satisfactory smoothness [15]. The corrosion resistance of burnished elements depends on the degree of deformation and smoothness of the surface. It is therefore important to correctly determine the parameters of burnishing depending on whether it is to be a smoothening or strengthening treatment.

The burnishing process, even though it was developed many years ago, is still used in many applications. The evidence of this is the number of publications devoted to this topic. This is indicated by the example of a review of publication databases such as scopus.com, which registered 403 publications on this topic in the years 2020–2024. The scope of use of burnishing technology is wide and allows it to be used for special alloys such as Inconel 718 [18,19] or aluminum alloys [20], not to mention duplex cast steels [5]. Currently, the problem of reducing the weight of structures makes it necessary to also look for new solutions for materials such as magnesium alloys [21,22]. For example, in paper [23], the authors employed mechanical burnishing, shot peening and ball burnishing to treat the surface of the high-strength magnesium alloy AZ80. They stated that the surface after ball burnishing not only obtained good fatigue performance but also showed superior corrosion fatigue properties in a 3.5% NaCl solution. Also, P. Zhang et al. [24] conducted tests on the AZ80 alloy and showed that, under a static pressure of 200 N, the fatigue strength of the alloy after the ball burnishing treatment increased by 110%.

Products are goods, objects and products that must be initially designed, then manufactured and finally sold, used and disposed of, or possibly reprocessed. Currently, efforts are being made to continuously improve and modernize manufacturing technologies in such a way as to meet the increasingly higher requirements of high-quality products from customers. Modern design of the product and its surface layer must be focused on construction integrated with production and then on operation. For example, burnishing treatment of the surface layer may be used, among other reasons, for the production and regeneration of pump drive shafts in place of seals.

## 2. Methodology of the Experimental Research

The aim of the research was to obtain satisfactory surface quality of the outer surfaces of cylinders (48 mm in diameter) made of corrosion-resistant X2CrNiMo17-12-2 steel. The assumption of the work was to carry out the burnishing process in such a way as to not only obtain a smoothing effect of the working surface after processing but also to potentially increase the service life of the component. The journals were prepared before burnishing by turning on a general-purpose TU250 × 1000 lathe. Turning parameters: cutting depth ap = 0.5 mm, feed rate f = 0.2 mm/rev, rotational speed *n* = 355 rpm and cutting speed vc = 55 m/min. To carry out slide burnishing, it is important to select optimal parameters and processing conditions. The feed rate should not be greater than the contact length of the burnishing tool with the workpiece because such a setting of the feed rate prevents the skipping of surface irregularities. The surface roughness in these omitted places would be the same as before machining. When setting the feed rate, special attention should be paid to the tool’s circular cross-section radius because this dimension is decisive for the tool contact length [8,10]. The second very important, perhaps the most important, parameter is the burnishing force, which is one of the factors responsible for the burnisher depth. To obtain the best smoothness, the tool should reach at least half of the height of the roughness ridges. Otherwise, the squashed ridges will not completely fill the valleys. In order to obtain better surface smoothness, the burnisher depth should be greater than half the height of the ridges. On the other hand, the penetration should not be deep, as this would create the headwave and the tailwave seen in plasticized metal. A less-than-optimal depth setting can produce new surface irregularities and the overall roughness may not be minimized. Another unwanted result of using too much force (i.e., going too deep into the material) can be an excessive crumpling of the surface layer, leading to such unfavorable effects as a decrease in the plasticity of the material and an increase in its internal stress [5,7]. If the burnishing parameter settings are suboptimal, the desired improvement in smoothness or hardness will not be achieved. This also has an economic dimension—repeating the process will increase the cost of machining. It is a good idea to perform this entire process in one pass of the tool over the surface of the workpiece because each subsequent step is less effective and, in addition, takes time. The feed rate and the burnishing force should be set in such a way that the squashed roughness ridges completely fill the valleys. The process of slide burnishing of the cylindrical surfaces was performed on a general-purpose TU250 × 1000 lathe, using a YAMATO YDB900652-003 slide burnisher (Sasso Marconi, Italy) with a rigid diamond-tipped clamp with a radius of 2.5 mm [11,17]. Based on the authors’ own research and recommendations provided in the YAMATO catalog [16], the following parameter values were used: burnisher depth an = 0.01–0.03 mm, burnishing force F = 30–90 N, feed rate f = 0.04–0.08 mm/rev, rotational speed *n* = 355 rpm and burnishing speed vn = 53 m/min. Additionally, corrosion-resistant steels, including X2CrNiMo17-12-2, are difficult to machine, so it was necessary to use machine oil to cool and lubricate the tools. The chemical composition of the steel samples was determined using a SOLARIS-CCD PLUS optical spark emission spectrometer (New Taipei City, Taiwan). It is presented in Table 1.

**Figure 3:**
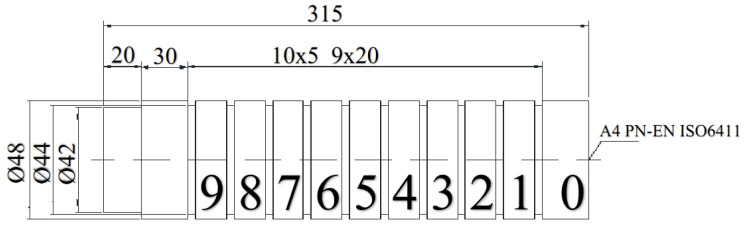
The arrangement of test samples with a diameter of 48 mm made of X2CrNiMo17-12-2 steel.

This composition meets the requirements of the standard [25]. The tests were carried out on a cylindrical sample made of X2CrNiMo17-12-2 steel with a diameter of 48 mm and other dimensions, as shown in Figure 3. Nine journals were made for testing in one pass, which prevented the occurrence of errors likely to be caused by machine reconfiguration. For each new sample, a completely new burnishing tool was used, so the tool was used only once. This approach was intended to eliminate potential defects caused by wear.

**Figure 4:**
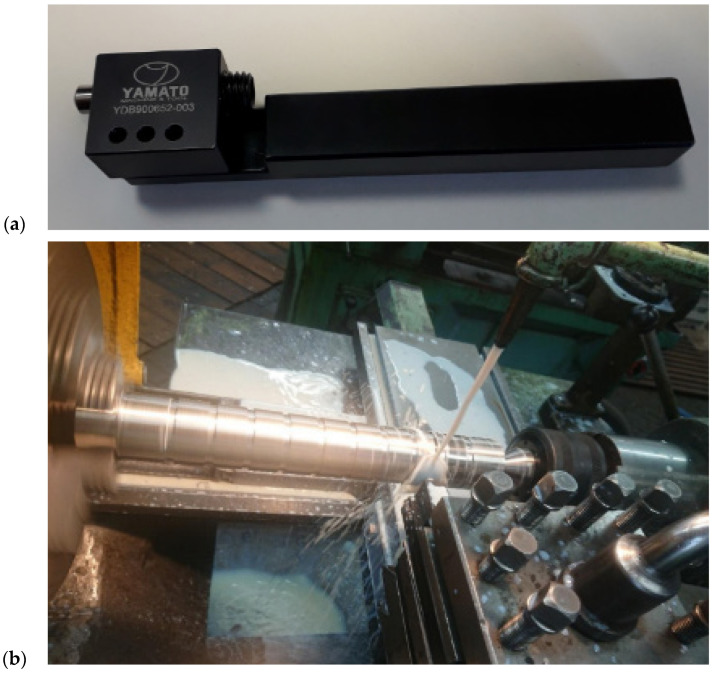
(**a**) YAMATO YDB900652-003 burnisher with a radius of 2.5 mm, and (**b**) the burnisher and shaft on the universal lathe.

The study measured the impact of changes in individual processing parameters on the properties of the machined material surface. The variable parameters in the study were the feed rate and the burnishing force. This force was measured using a static FT-5304M/A/16 strain gauge load meter. It was determined that 1 mm of burnisher depth corresponds to 3 kN of burnishing force. The slide burnishing was carried out using a YAMATO YDB900652-003 tool on a general-purpose TU250 × 1000 universal lathe (Figure 4).

**Figure 5:**
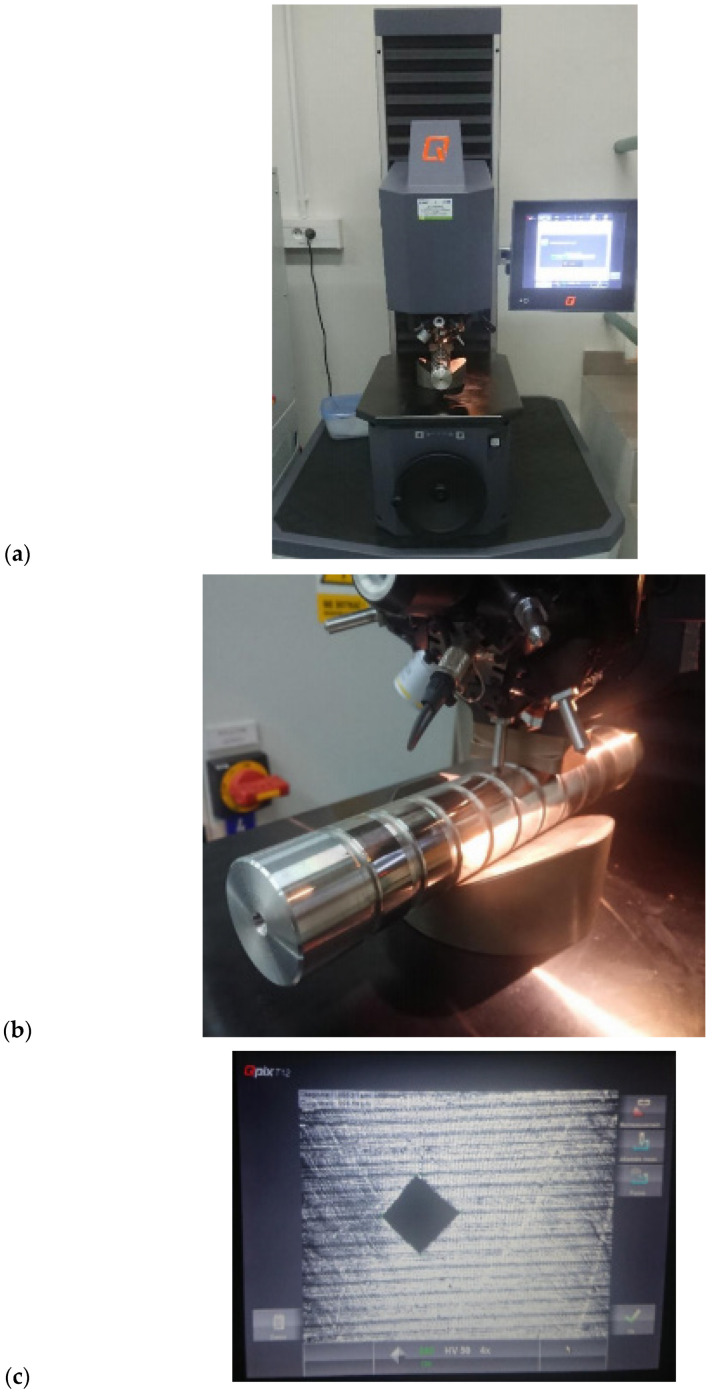
Qness Q250M hardness tester: (**a**) general view, (**b**) view during measurement, (**c**) surface with visible indent.

A Qness Q250M hardness tester was used to measure hardness (Figure 5). It allows for very accurate and fully automatic measurement using the Vickers, Brinell, Rockwell and Knoop methods. The hardness measurement was performed using the Vickers method according to the standard [26] for a burnishing force of 50 N at three measurement points on each journal.

**Figure 6:**
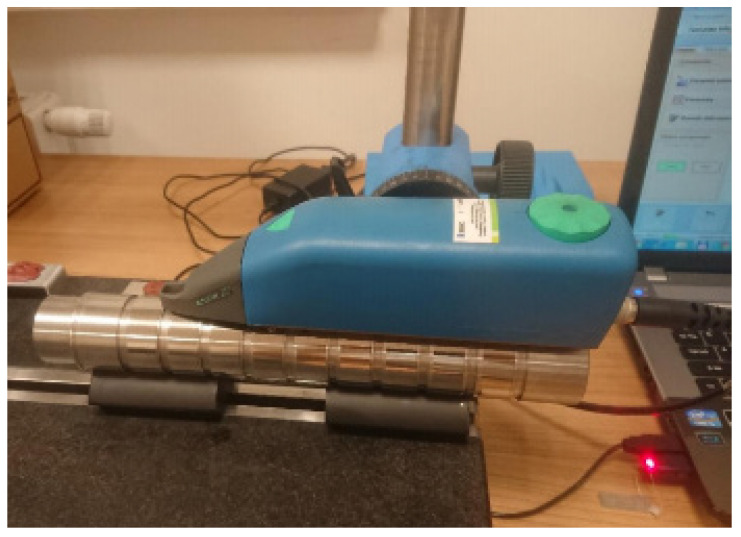
HOMMEL-ETAMIC W20 roughness profilometer.

The roughness measurement was performed using a HOMMEL-ETAMIC W20 profilometer (Villingen-Schwenningen, Germany) with a TKL 300 L head (Figure 6) in accordance with the standard [27] for a measuring section of 4.8 mm and for an elementary section of 0.8 mm. The arithmetic mean of the profile deviation from the mean line (Ra) and the roughness ridge height according to ten profile points (Rz) were measured. The measurement was performed at four points on each journal.

## 3. Results of Experimental Research

The aim of the experimental research was to determine the influence of burnishing parameters and conditions on the surface roughness and hardness of the surface layer of corrosion-resistant steel. Table 2 presents the process parameters and results of the roughness measurements after burnishing (pin 0 after turning, pins 1–9 after burnishing).

**Figure 7:**
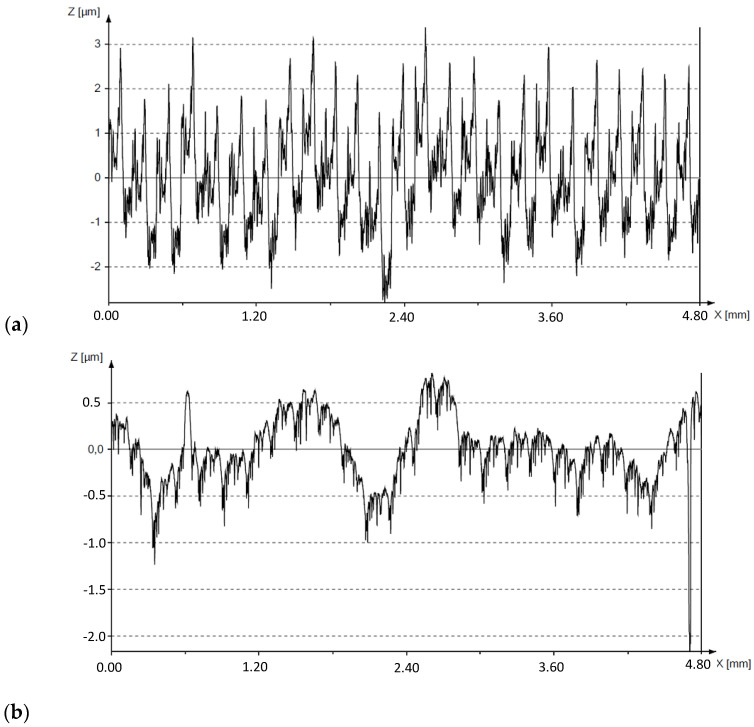
Examples of surface roughness profiles plotted by the HOMMEL-ETAMIC W20 device: (**a**) after turning, (**b**) after slide burnishing.

Figure 7 shows examples of surface roughness profiles plotted by the HOMMEL-ETAMIC W20 profilometer after turning and after slide burnishing.

Based on the analysis of the test results presented in Figure 7, it can be seen that burnishing significantly reduced the surface roughness. The results show that slide burnishing was carried out correctly, as the intention was to reduce the roughness. Also, the results of the calculations performed using the equations [5,11] were analyzed:

(1) $$K_{Ra}=\frac{R^{\prime }a}{Ra}$$

(2) $$K_{Rz}=\frac{R^{\prime }z}{Rz}$$

where

*R*′*a*, *R*′*z*—roughness after turning;
*Ra*, *Rz*—roughness after slide burnishing.

**Figure 8:**
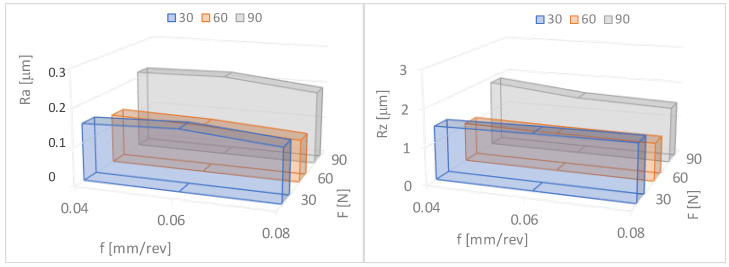
Dependence of surface roughness on the burnishing force (F) and the feed rate (f). Ra—the arithmetic mean roughness value from the amounts of all profile values, Rz—maximum height of profile average value of the five measurements.

Figure 8 shows the dependence of the surface roughness Ra and Rz on the change in the feed rate and the burnishing force. Within the given force range, it can be seen that as the burnishing force increases, the surface roughness value decreases. The lowest values of Ra and Rz were obtained when the burnishing force F = 90 N and the feed rate f = 0.06 mm/rev. When the feed rate f = 0.08 mm/rev, the values of Ra and Rz after slide burnishing were the highest.

**Figure 9:**
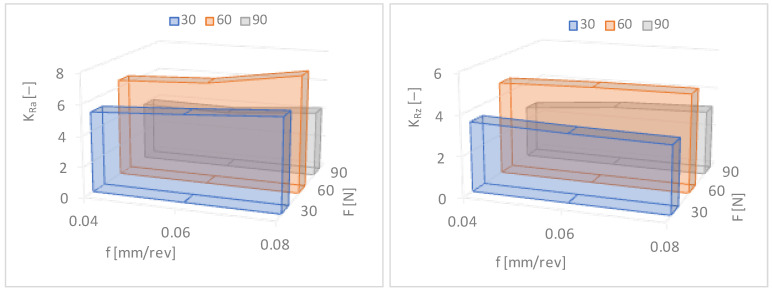
Dependences of the surface roughness reduction rates (KRa—arithmetic mean roughness value from the amounts of all profile values, KRz—maximum height of profile average value of the five measurements) on the feed rate (f) and the burnishing force (F).

The surface roughness reduction rates determined using Formulas (1) and (2) are presented in Table 2 and Figure 9. It can be seen (Figure 9) that with an increase in force in the range of F = 30–90 N and the feed rate f = 0.04–0.06 mm/rev, the roughness reduction rate for Ra and Rz increases. The lowest values of Ra = 0.11 μm and Rz = 1.01 μm, and the greatest reduction in surface roughness KRa = 7.64 and KRz = 4.85, were obtained for the feed rate f = 0.06 mm/rev and the burnishing force F = 90 N.

Table 3 shows the hardness measurement results after slide burnishing. The degree of relative strengthening of the surface layer was determined by the following equation [5,11]:

(3) $$S_{u}=\frac{HV-HV^{\prime }}{HV^{\prime }}\;100\%$$

where

*HV*′, *HV*—surface layer hardness before and after burnishing

As can be seen from Table 3, an increase in hardness was observed. This increase is up to approximately 20 Vickers units. Although this increase is not significant, it guarantees less friction in combination with the reduction in roughness. It should therefore be concluded that slide burnishing extends the service life of the working surface by not only reducing the surface roughness but also by increasing its hardness. The highest degree of relative hardening, Su = 7.61%, occurs when the feed rate f = 0.06 mm/rev and the burnishing force F = 90 N. The smallest increase in the hardness of the surface layer occurs for the feed rate f = 0.08 mm/rev. The degree of relative strengthening is several percent.

**Figure 10:**
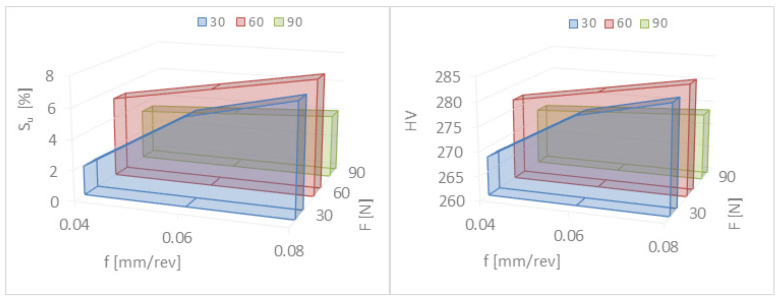
Dependence of the degree of relative strengthening (Su) and hardness (HV) on the burnishing force (F) and the feed rate (f).

From Table 3 and Figure 10, it can be seen that the increase in hardness was not significant but occurred in every journal (in every case). The research methodology and selection of technological parameters for burnishing treatment were carried out on the basis of the literature [28,29,30] and our own previous research. Increasing hardness along with reducing surface roughness also improved the resistance parameters to tribological wear, especially in the case of sliding friction in the presence of lubricant. Sliding burnishing is used as an anti-fatigue and anti-friction treatment, as well as for smoothness, in place of polishing and grinding, mainly due to lower burnishing costs and also due to a less harmful impact on the environment.

This indicates that slide burnishing not only reduces the surface roughness but also hardens the surface layer of the material. As the burnishing force increases in the range of F = 30–90 N, the hardness of the surface layer of steel increases. The highest degree of relative hardening, Su = 7.61%, occurs when the feed rate f = 0.06 mm/rev and the burnishing force F = 90 N. The smallest increase in the hardness of the surface layer occurs for the feed rate f = 0.08 mm/rev. The degree of relative strengthening is several percent. This is not a very large increase in hardness but it is visible and has a significant impact on the operational wear of machine elements. However, slide burnishing is used in particular for smoothing purposes.

**Figure 11:**
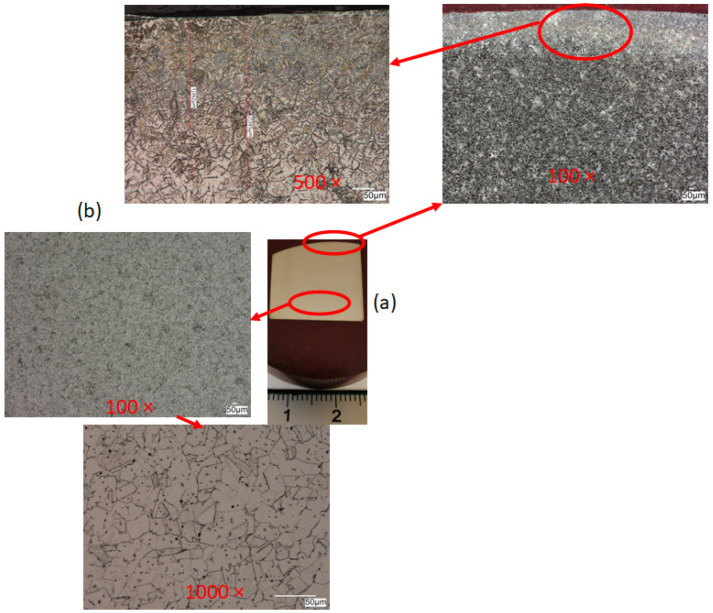
Cross-sectional view after burnishing: (**a**) macroscopic sample view, (**b**) microscopic observation.

**Figure 12:**
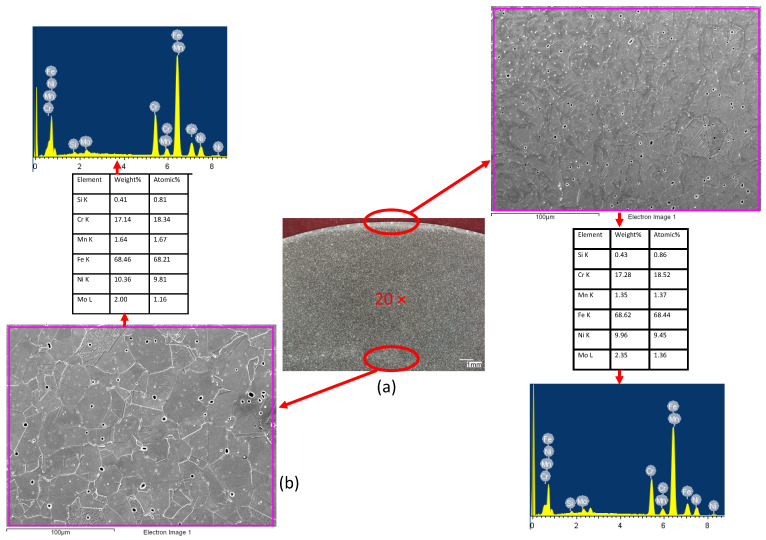
Cross-sectional view after burnishing: (**a**) macroscopic observation, (**b**) microscopic observation.

After experimental tests of the slide burnishing of cylindrical elements made of corrosion-resistant steel X2CrNiMo17-12-2, it can be concluded that a significant reduction in surface roughness and an increase in the degree of relative strengthening of the material occurs for the feed rate in the range of f = 0.04–0.06 mm/rev and the burnishing force in the range of F = 30–90 N. Figure 11 shows the results of macroscopic and microscopic observations after burnishing. For convenience, the observation areas are marked in red (Figure 11 and Figure 12).

Deformations in the surface layer are clearly visible and their measured depth is 400 μm. A clear grain orientation can be seen in the surface area, i.e., the area subjected to the plastic forming process. The deformed elongated grains have the same orientation with homogenous mechanical properties in the surface layer, which is particularly important in terms of the potentially negative impact of the burnishing process, as described by the authors of publications [5,11,13,17], among others. The core area is characterized by a clearly equiaxed microstructure and high grain size uniformity. One of the advantages of the burnishing process, apart from the relatively low cost [31,32,33], is the fact that there is no change in the chemical composition of the material [34,35,36,37]. Therefore, the authors performed scanning electron microscopy with energy-dispersive X-ray spectroscopy (SEM/EDX) to confirm this fact. Figure 12 shows the results of the analysis of the deformed surface and core areas.

As can be seen, the chemical composition is similar in both areas, which indicates that the burnishing process ran correctly, without contamination of the material surface with solids from the burnisher. Grains clearly deformed in the direction of the application of force are visible in the area of the surface layer. No cracks or tears were observed in this area, which also indicates that the proposed parameters are correct. The core is characterized by an equiaxed microstructure.

## 4. Discussion

The article describes the influence of changes in burnishing conditions and parameters—such as the feed rate and the burnisher depth (pressure force) at a constant burnishing speed—on the surface roughness and hardness of the surface layer of the material [38,39,40]. Modern design of the product and its surface layer must be focused on construction integrated with production and then on operation. Burnishing of the surface layer may be used, among other techniques, for the production and regeneration of pump drive shafts in place of seals, crankshaft journals, pins and intake and outlet valves. The main mechanism influencing hardness in the burnishing process is strengthening. Due to the fact that the process is carried out cold, the temperature increase in the contact zone is negligible, not more than about 25 degrees (based on the authors’ previous research), and there are no microstructure reconstruction phenomena. This is clearly visible in the form of elongated grains in the direction of deformation. Of course, during plastic deformation during burnishing, the same phenomena occur in the surface layer as during cold rolling.

Burnishing offers many possibilities like reducing the surface roughness, but also, the machined object becomes more resistant to abrasion and has better resistance to fatigue [41,42,43]. The surface layer is squashed during burnishing, while the deeper layers of the material are not affected. As a result, the hardness on the surface of the workpiece increases, but the material still retains its properties. The main aim of this work was to investigate the influence of changes in burnishing parameters on the final quality of the machined surface. The focus was on parameters such as the feed rate and the burnishing force, and the surface was checked for changes in its roughness and hardness. It can be concluded from the results of the study that the changed parameters have a significant impact on the quality of the machined surface [44,45,46]. Changes in the feed rate have a very strong impact on the roughness of the surface layer, but its impact on hardness is much smaller. It can be noticed that increasing the feed rate causes minor changes up to a certain point, but after exceeding it, the surface roughness increases dramatically. This is caused by the burnishing tool leaving part of the surface unprocessed. In turn, the infeed force is the opposite—when changing the roughness of the workpiece, it has a small effect, but changes the hardness of the surface layer to a much greater extent. With the increase in the feed force, the final hardness increases, but it cannot be increased indefinitely. One of the limitations is the tailwave formed behind the tool, which negatively affects the final smoothness. Another cause is that too much pressure on the diamond tip can accelerate its wear, which will significantly increase the cost of processing. It follows that the selection of burnishing parameters depends on the purpose for which we use this treatment. The main parameter for smoothness is the feed rate. In order to obtain a specific hardness, an appropriate burnishing force must be selected. Because small feed rates and burnishing forces are recommended for slide burnishing with a rigid diamond-tipped clamp, their values were set at f = 0.04–0.08 mm/rev and F = 30–90 N, respectively. After analyzing the test results for slide burnishing of outer cylindrical surfaces made of corrosion-resistant steel X2CrNiMo17-12-2, it was found that the greatest reduction in surface roughness (KRa = 7.64 and KRz = 4.85) and the greatest relative degree of material strengthening (Su = 7.61%) occurred for the feed rate f = 0.06 mm/rev and the burnishing force F = 90 N. Therefore, in order to obtain the appropriate quality of cylindrical elements made of corrosion-resistant steel X2CrNiMo17-12-2, it seems reasonable to use a feed rate in the range of f = 0.04–0.06 mm/rev and a burnishing force in the range of F = 30–90 N. The selection of the technological process parameters used should depend on the purpose of the burnishing treatment being used. The technological process aimed at obtaining low surface roughness should be carried out with the greatest possible force pressing the working element to the processed surface, while the burnishing speed and feed should be as low as possible. The lowest values of the Ra and Rz parameters occur when a burnishing force of 90 N and a feed rate of 0.06 mm/rev are applied. When using a feed of 0.08 mm/rev, the values of these roughness parameters after slide burnishing are the highest. This is due to the fact that at low forces (30 N), the feed is greater than the contact length and a small recess occurs, and thus, the roughness is greater than when the lower feed is used (0.04–0.06 mm/rev). However, when using high force values (90 N) and high feed values (0.08 mm/rev), the feed is smaller than the contact length with a large tool cavity. A so-called wave is created in front of the burnishing element, and therefore, the roughness is greater compared to the same force (90 N) but at a lower feed (0.04–0.06 mm/rev). Experimental tests have confirmed the possibility of improving some functional properties, including reducing roughness and increasing hardness, by strengthening the surface layer. A surface with a low roughness shows greater resistance to corrosion than a surface with a higher roughness, with the same chemical composition. However, the surface layer with higher hardness and at the same time low roughness, according to the literature data [10,22,43], should be characterized by higher resistance to tribological wear while maintaining high fatigue strength.

## 5. Conclusions

Based on the research and obtained results, the following conclusions could be formulated:

- The burnishing process can provide a final roughness similar to that produced by smooth finishing.
- The microstructure, or rather the grains, in the area of the surface layer is oriented in the direction of deformation.
- The analysis of the microstructure in the area of the surface layer did not reveal any cracks or other flaws, which proves that the recommended process is correct.
- The slide burnishing not only reduces the surface roughness but hardens the surface layer of the burnished material.
- The greatest reduction in the surface roughness (KRa = 7.64 and KRz = 4.85) and the greatest relative degree of material strengthening (Su = 7.61%) occur for the feed rate f = 0.06 mm/rev and the burnishing force F = 90 N.
- It is best to use a burnishing feed rate in the range of f = 0.04–0.06 mm/rev and a burnishing force in the range of F = 30–90 N.
- The use of a feed rate of 0.08 mm/rev increased the surface roughness; the feed is smaller than the contact length with a large tool cavity, and a so-called wave is created in front of the burnishing element.

## Figures and Tables

**Table 1:** Chemical composition of corrosion-resistant steel X2CrNiMo17-12-2.

C [%]	Cr [%]	Ni [%]	Mo [%]	Cu [%]	Mn [%]	Si [%]	S [%]	P [%]	Nb [%]	Co [%]	V [%]	W [%]
0.024	16.449	9.286	2.058	0.544	0.946	0.381	0.024	0.019	0.03	0.218	0.091	0.022

**Table 2:** Results of roughness measurements and indicators of roughness reduction after turning and burnishing with the parameters of slide burnishing.

Nr	F [N]	f [mm/rev]	Rz [μm]	Ra [μm]	K_Ra_ [−]	K_Rz_ [−]
0	-	0.2	4.92	0.84	-	-
1	30	0.04	1.43	0.16	5.25	3.44
2	60	0.04	1.48	0.15	5.60	3.32
3	90	0.04	1.51	0.14	6.00	3.26
4	30	0.06	1.05	0.13	6.46	4.66
5	60	0.06	1.02	0.12	6.72	4.80
6	90	0.06	1.01	0.11	7.64	4.85
7	30	0.08	1.85	0.21	3.91	2.66
8	60	0.08	1.60	0.22	3.81	3.07
9	90	0.08	1.52	0.20	4.25	3.23

**Table 3:** The degree of relative strengthening and hardness after slide burnishing.

Nr	F [N]	F [mm/obr]	S_u_ [%]	HV	Stand. Dev. for HV
0	-	0.20	-	263	1.49
1	30	0.04	1.91	268	0.96
2	60	0.04	5.71	278	1.60
3	90	0.04	7.22	282	1.71
4	30	0.06	5.32	277	1.29
5	60	0.06	6.46	280	1.80
6	90	0.06	7.61	283	2.50
7	30	0.08	3.42	272	2.69
8	60	0.08	3.81	273	2.99
9	90	0.08	4.18	274	3.14

## Data Availability

Data are contained within the article.
